# Supplementary material for: The Benefits of Using Saccharose for Photocatalytic Water Disinfection
Source: Int J Mol Sci. 2022 Apr 25;23(9):4719. doi: 10.3390/ijms23094719 (PMC9101646; doi:10.3390/ijms23094719)
Supplement: Supplementary file 1 [file ijms-23-04719-s001.zip › ijms-1685355-supplementary.pdf]

Supplementary Materials

The Benefits of Using Saccharose for Photocatalytic Water Disinfection

Paulina Rokicka-Konieczna[1]*, Agata Markowska-Szczupak[2], Ewelina Kusiak-Nejman[1]; Antoni W. Morawski[1]

[1] Department of Inorganic Chemical Technology and Environment Engineering, Faculty of Chemical Technology and Engineering, West Pomeranian University of Technology in Szczecin, Pułaskiego 10, 70-322 Szczecin, Poland; ewelina.kusiak@zut.edu.pl (E.K.-N.); antoni.morawski@zut.edu.pl (A.W.M.)

[2] Department of Chemical and Process Engineering, Faculty of Chemical Technology and Engineering, West Pomeranian University of Technology in Szczecin, Al. Piastów Ave. 42, PL 71-065 Szczecin, Poland; (A.M.-S.);

* Correspondence: paulina.rokicka@zut.edu.pl (P.R.-K) , tel. +48 091 449 47 30

SM 1. R^2 value obtained from the kinetic analyses

Table S1. R^2 value obtained from the kinetic analyses of four model for *E. coli* under UV-A irradiation.

E. coli UV-A	R^2 value			
Samples	Chick-Watson	Modified Chick-Watson	Hom	Modified Hom
KRONOClean7000	0.923	0.925	0.982	0.989
TiO_2-100	0.921	0.919	0.995	0.995
TiO_2-S-1%-100	0.787	0.786	0.948	0.965
TiO_2-S-5%-100	0.775	0.769	0.987	0.991
TiO_2-S-10%-100	0.847	0.815	0.960	0.979

Table S2. R^2 value obtained from the kinetic analyses of four model for *E. coli* under ASL irradiation.

E. coli ASL	R^2 value			
Samples	Chick-Watson	Modified Chick-Watson	Hom	Modified Hom
KRONOClean7000	0.872	0.873	0.981	0.998
TiO_2-100	0.817	0.815	0.944	0.948
TiO_2-S-1%-100	0.703	0.765	0.889	0.909
TiO_2-S-5%-100	0.933	0.934	0.990	0.998
TiO_2-S-10%-100	0.771	0.769	0.955	0.979

Table S3. R^2 value obtained from the kinetic analyses of four model for *S. epidermidis* under UV-A irradiation.

S. epidermidis UV-A	R^2 value			
Samples	Chick-Watson	Modified Chick-Watson	Hom	Modified Hom
KRONOClean7000	0.917	0.963	0.973	0.977
TiO_2-100	0.891	0.949	0.990	0.993
TiO_2-S-1%-100	0.825	0.816	0.971	0.984

TiO_2-S-5%-100	0.856	0.854	0.980	0.982
TiO_2-S-10%-100	0.745	0.743	0.957	0.965

Table S4. R^2 value obtained from the kinetic analyses of four model for *S. epidermidis* under ASL irradiation.

S. epidermidis ASL	R^2 value			
Samples	Chick-Watson	Modified Chick-Watson	Hom	Modified Hom
KRONOClean7000	0.887	0.887	0.967	0.969
TiO_2-100	0.863	0.861	0.986	0.999
TiO_2-S-1%-100	0.674	0.673	0.930	0.969
TiO_2-S-5%-100	0.794	0.787	0.954	0.963
TiO_2-S-10%-100	0.655	0.648	0.923	0.958

SM 2. Kinetic constants obtained from the kinetic analyses

Table S5. Kinetic constants obtained from the kinetic analyses of four model for *E. coli* under UV-A irradiation.

E. coli UV-A	Equation							
Samples	Chick-Watson	Modified Chick-Watson		Hom		Modified Hom		
	k	k_1	k_2	k	h	k_1	k_2	k_3
KRONOClean7000	0.020603	63.1303	0.000329	0.002336	1.532227	2.57612	0.030191	3.898640
TiO_2-100	0.023618	82.0273	0.000290	0.001165	1.770434	65.1624	0.002447	1.822669
TiO_2-S-1%-100	0.021202	83.1971	0.000256	0.000017	2.950902	259.743	0.003640	3.015055
TiO_2-S-5%-100	0.018975	24.2523	0.000791	0.000010	2.912336	228.103	0.003775	3.131825
TiO_2-S-10%-100	0.018891	66.0274	0.000288	0.000254	2.081988	112.064	0.002193	2.140370

Table S6. Kinetic constants obtained from the kinetic analyses of four model for *E. coli* under ASL irradiation.

E. coli ASL	Equation							
Samples	Chick-Watson	Modified Chick-Watson		Hom		Modified Hom		
	k	k_1	k_2	k	h	k_1	k_2	k_3
KRONOClean7000	0.016305	61.1268	0.000268	0.000100	2.206869	94.6816	0.002292	2.298026
TiO_2-100	0.010709	45.7506	0.000236	0.000013	2.585416	96.3337	0.002623	2.704722
TiO_2-S-1%-100	0.013317	55.2116	0.000224	0.000016	2.634772	70.9731	0.003694	2.778838
TiO_2-S-5%-100	0.014419	47.3373	0.000307	0.000959	1.166276	47.1840	0.001680	1.703885
TiO_2-S-10%-100	0.014232	62.8195	0.000228	0.000001	3.233536	57.7150	0.006521	4.031804

Table S7. Kinetic constants obtained from the kinetic analyses of four model for *S. epidermidis* under UV-A irradiation.

S. epidermidis UV-A	Equation

Samples	Chick-Watson	Modified Chick-Watson		Hom		Modified Hom		
	k	k_1	k_2	k	h	k_1	k_2	k_3
KRONOClean7000	0.019263	60.6838	0.000323	0.002089	1.543072	2.80770	0.024043	3.178398
TiO_2-100	0.018569	44.0106	0.000499	0.000543	1.873808	55.5178	0.002601	1.996487
TiO_2-S-1%-100	0.016223	13.3154	0.001220	0.000057	2.438614	131.908	0.002891	2.544923
TiO_2-S-5%-100	0.015807	58.7598	0.000270	0.000184	2.117424	102.138	0.002199	2.181630
TiO_2-S-10%-100	0.012176	59.9702	0.000204	0.000008	2.800929	144.689	0.003165	2.800929

Table S8. Kinetic constants obtained from the kinetic analyses of four model for *S. epidermidis* under ASL irradiation.

S. epidermidis ASL	Equation							
Samples	Chick-Watson	Modified Chick-Watson		Hom		Modified Hom		
	k	k_1	k_2	k	h	k_1	k_2	k_3
KRONOClean7000	0.015497	267.181	0.000058	0.000298	1.925855	76.6035	0.001669	1.950244
TiO_2-100	0.015254	66.9831	0.000244	0.000061	2.321625	100.188	0.002519	2.439041
TiO_2-S-1%-100	0.014330	68.0058	0.000211	0.000001	5.357898	30.5245	0.021755	12.25727
TiO_2-S-5%-100	0.011225	12.8729	0.000890	0.000006	2.790017	104.386	0.003302	3.080018
TiO_2-S-10%-100	0.008807	44.8321	0.000206	0.000002	4.529654	311.576	0.005021	5.283768

SM 3. Emission spectra of light sources

a)

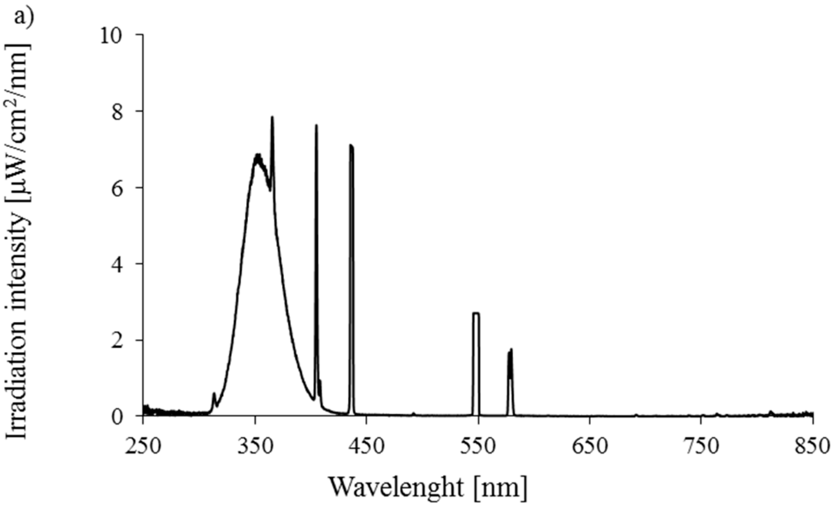

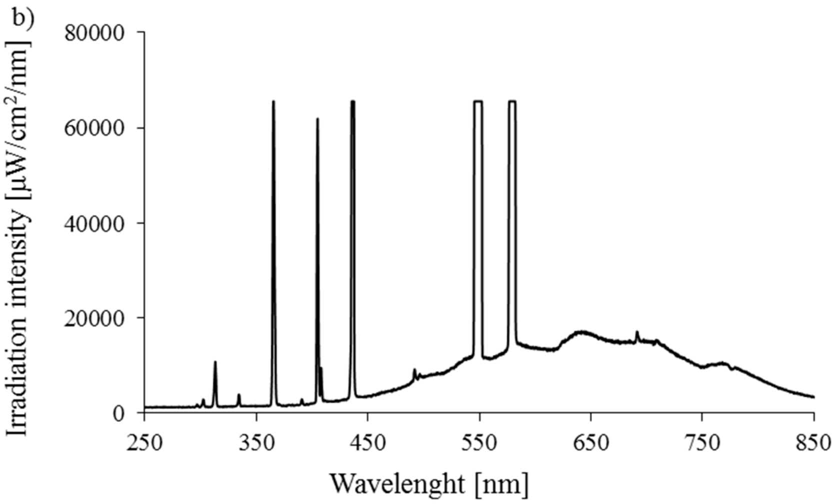

Figure S1. Emission spectra of light sources: a) UV-A, b) artificial solar light (ASL).
